# Barriers and Facilitators to Seeking Sleep Solutions for Children With Cerebral Palsy: A Qualitative Study

**DOI:** 10.3389/fpsyt.2021.729386

**Published:** 2021-11-17

**Authors:** Sacha Petersen, Dinah S. Reddihough, Sally Lima, Adrienne Harvey, Fiona Newall

**Affiliations:** ^1^School of Health and Biosciences, RMIT University, Bundoora, VIC, Australia; ^2^University of Melbourne, Parkville, VIC, Australia; ^3^Royal Children's Hospital, Parkville, VIC, Australia; ^4^Murdoch Children's Research Institute, Parkville, VIC, Australia; ^5^Bendigo Health, Bendigo, VIC, Australia

**Keywords:** sleep, children, cerebral palsy, health services, qualitative study

## Abstract

**Background:** Published evidence to date suggests that sleep problems are common in children with cerebral palsy (CP). This qualitative study is a follow up to a previously published quantitative phase on the experience and impact of sleep problems in this population.

**Aims:** The aim of this study was to explore the experience and impact of sleep disturbance and seeking of sleep solutions for parents of school aged children with CP.

**Materials and Methods:** Semi-structured 19 qualitative interviews were conducted with parents of children with CP aged 6–12 years. Interview data were transcribed verbatim and the thematic analysis techniques by Braun and Clarke was used to identify themes.

**Results:** Thematic analysis identified 7 themes: (1) *My Child Doesn't Fit into the Box*, (2) *A Mother's Ears are Always On*, (3) *Sleep Disturbance is like Water Torture*, (4) *Sleep is One of Many Spot Fires, I Put it on the Backburner*, (5) *Luck, Money or Jumping Up and Down*, (6) *There is Never One Silver Bullet* and (7) *Help: The Earlier the Better*. The key finding was that parents of children with CP often described their child's needs being distinct from what is provided by systems and services.

**Conclusion:** Parents face significant challenges sourcing effective sleep solutions for their child with CP. Sleep is often not a priority for either the parent or the clinician as other health problems take precedence. Parents reflected that early sleep intervention for their child was or would have been helpful. The barriers and facilitators to sleep care identified in this study should be used to inform clinical change in care for children with CP. Sleep needs to be prioritized in healthcare for children.

## Introduction

Cerebral palsy (CP) describes:

*“A group of permanent disorders of the development of movement and posture, causing activity limitations, attributed to non-progressive disturbances that occurred in the developing fetal or infant brain. The motor disorders of cerebral palsy are often accompanied by disturbances of sensation, perception, cognition, communication and behaviour, epilepsy, and by secondary musculoskeletal problems”* [(1), p. 9].

Sleep problems are common in school aged children with CP and can have significant impact on the child and their parents. Parents of children with a disability report poor sleep (2–4) and that chronic sleep disturbance results in a negative impact on parent's health and well-being (3–5) including reduced subjective health (4), reduced participation in healthcare activities (4), stress (2, 3), depression (3), anxiety (3) and reduced quality of life (5). Studies (6–13) that have compared the sleep of children with CP to their typically developing peers have found the incidence of sleep problems is significantly higher for children with CP. A Malaysian study (6) showed only 5% of typically developing children had a pathological sleep score, while 30% of their siblings with CP scored within the pathological range on a sleep-screening tool, of particular importance as surveying siblings removed the variance of culture and parenting style. This study was supported by more recent research (14). There are many studies that have explored the frequency and reasons for sleep problems in this cohort (12, 15–25). Positioning, pain, seizures and continence are the most commonly reported care and comfort reasons for sleep disturbance in addition to an association between pathological sleep scores with epilepsy (191, 192), single parent households (24) and poorer psychological health for parents (26). It is important to recognize that studies which limit their data collection to the commonly used validated sleep assessment tools, such as the Children's Sleep Habit Questionnaire (CSHQ) (27) and the Sleep Disturbance Scale for Children (SDSC) (28), will produce results that align with those tools and are broad descriptors of sleep problems. Whilst this broad data is important, it does not consider the complexity of sleep problems or the context of how families seek sleep care for children with neurodisability. There is a paucity of research that explores why sleep issues are so frequent in children with CP or the barriers and facilitators to accessing effective care in this population.

This paper presents the findings of the third of three-phase exploratory sequential mixed methods study. Phase 1 of this study was a scoping phase, via exploratory interviews, with parents of children with CP who had sleep problems; this phase informed the design of a quantitative survey. The second phase of this study was a quantitative survey, including validated sleep screening tools. The first two phases of the study have been described in detail in a previously published paper (25). The aim of this qualitative phase was to explore the experience and impact of sleep disturbance and how parents sought solutions to these problems.

## Materials and Methods

All participants were parents or primary caregivers of children with CP aged 6–12 years. Participants were purposively sampled from the quantitative Phase 1 of the study (25). Sampling included choosing participants based on their experience of sleep problems: the best and worst sleepers and the severity of their child's CP according to their Gross Motor Function Classification System (GMFCS) level (29). The GMFCS is an internationally recognized method of describing a child's gross motor function using one of five levels. Children classified within GMFCS level I can walk independently with mild gait disturbance, whilst children with CP classified within GMFCS level V experience severe motor impairment, are unable to independently ambulate or have poor head control (30). Participants who met these inclusion criteria and agreed to further contact were invited to interview. The recruitment process was performed in rounds to ensure that there was time to conduct and transcribe each interview before moving on to the next group of participants. This allowed for iterative analysis; data from each round informed subsequent interviews.

Semi structured interviews were conducted face-to-face or over the phone. The interview tool was designed based on the analysis of the previous two phases of the study. In addition to this tool, the information taken from the participant's survey responses was also used as a prompt for questioning.

Interviews were audio recorded and were transcribed verbatim. Participants were not asked to review the interview transcripts, the additional request of time to review their interview may have acted as a deterrent for participation, given the difficulties in recruiting participants and the research fatigue reported by them in previous phases. Inductive thematic analysis, was undertaken (99) following the six phases outlined by Braun and Clarke (31); (1) Familiarizing yourself with your data: (2) Generating initial codes: (3)Searching for themes: (4) Reviewing themes: (5) Defining and naming themes: (6) Producing the report. Authors SP and SL read and re-read the interview transcripts. SP completed the initial coding of the data, SL independently coded the data and both authors convened to decide on final codes to be used. NVivo (32) software was utilized to assist with data management and coding. Steps three through to five of the thematic analysis occurred as an iterative process between authors SP and SL. Step six was performed by author SP and edited by author SL.

Throughout the data collection and analysis phase peer debriefing, and reflexivity was used to minimize bias on behalf of the researchers.

Ethics approval was obtained from The Royal Children's Hospital, Melbourne Human Research and Ethics Committee (HREC #37300).

## Results

Recruitment occurred from August 2018 to March 2019. A total of 19 parents were recruited and interviewed. Participant details can be found in Table 1. The participants were a diverse group of parents and children, representative of the heterogeneity that occurs within the CP population. The group consisted of mostly mothers, with three participants being fathers. Notably, of the seven who had a “good” sleep score, five had some experience with poor sleep even if they currently reported no sleep problems. This meant that interviews with those who had no current sleep problems contained rich and informative data about the experience of sleep problems and what had been done to improve them.

**Table 1:** Participant demographics and interview details.

**Pseudonym**	**Relationship to child**	**Age of child**	**GMFCS level**	**CSHQ score**	**Family type**	**Interview format** ***P* = Phone** **I = In person**	**Interview** **length** **(minutes)**	**Type of sleep problems (child)**
Vela	Mother	12	IV	44	Partnered, 3 children	P	84	Currently sleeps well. Long history of sleep problem. Sleep latency and frequent wakings
Ursa	Mother	11	V	58	Blended family, 4 children	P	60	Multiple sleep problems related to comorbidities of CP
Halley	Mother	10	II-III	34	Partnered, 4 children	P	30	Good sleep now, did have poor sleep prior to starting school
Juliet	Mother	10	V	36	Partnered, 2 children	P	32	Currently sleeps ok, occasionally wakes. Long history of night-time wakings and screaming at night
Atlas	Father	7	II	38	Partnered, 2 children	P	32	Sleeps well. History of mild sleep latency
Bianca	Mother	7	II	44	Partnered, 3 children	I	56	Sleeps well mostly, always has
Alya	Mother	12	II	36	Partnered, 2 children	P	38	Sleeps well now, some issues with anxiety and sleep latency. Has found strategies that work
Lyra	Mother	12	III	41	Partnered, 3 children	P	33	No sleep problems
Cressida	Mother	12	I	37	Partnered, 3 children	P	42	No sleep problems
Cordelia	Mother	12	I	53	Single, 4 children	P	60	Restless nights and often sleep latency
Orion	Father	13	I	45	Partnered, 2 children	P	22	Good sleeper, no history of sleep problems
Elara	Mother	8	V	48	Partnered, 1 child	P	63	Sleep problems related to comorbidities of severe CP – tone and discomfort
Ariel	Mother	10	II	46	Partnered, 3 children	P	49	Night-time waking and anxiety
Phoebe	Mother	8	III	53	Partnered, 2 children	P	50	Night-time waking and bed time separation anxiety
Ophelia	Mother	11	III-IV	60	Partnered, 3 children	P	70	Co-sleeping. Significant attachment frequent night waking
Pandora	Mother	13	II	46	Partnered, 2 children	P	26	No current sleep problems. History of significant sleep problems as an infant
Portia	Mother	13	V	51	Partnered, 2 children	I	32	Sleeps well, requires repositioning and care overnight
Titania	Mother	10	II	52	Partnered, 2 children	P	40	Long periods awake overnight
Norma and Leo	Mother and Father	7	V	47	Partnered, 3 children	I	27	Light sleeper, but generally sleeps well

Of the 19 interviews, 12 were parents of children who scored over 41 on the CSHQ (indicating likely poor sleep) and seven scored 41 or less on the CSHQ (indicating no sleep problems); according to the previous phase survey results. Only three parents reported that their child had no current problems and had never had any sleep problems. That is, 16 of the 19 participants had experienced sleep problems in their child. In the instances where parents had no experience with sleep problems, they were asked about their experience of accessing healthcare for other health problems.

**Figure 1:**
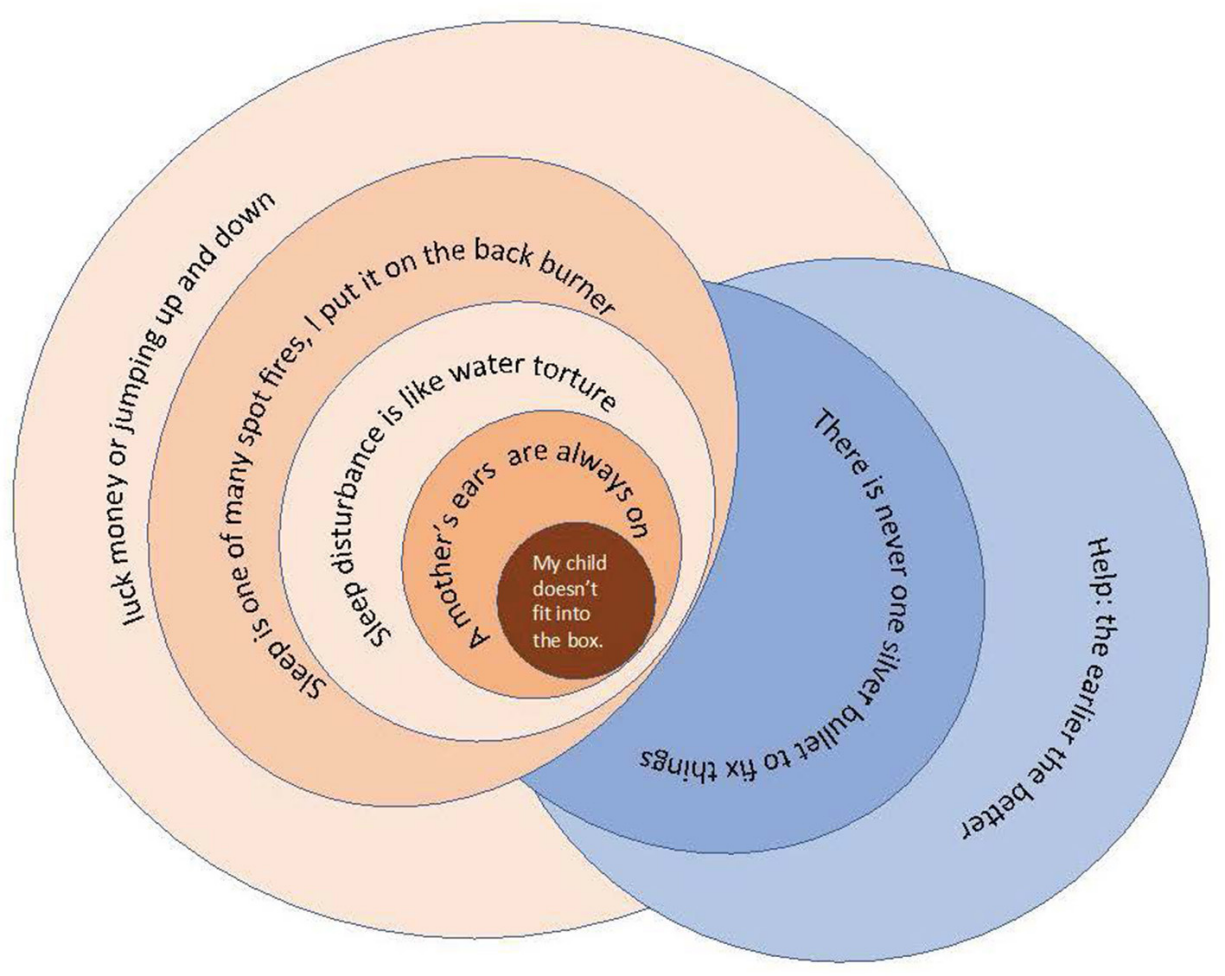
Concept map of the thematic analysis.

The themes identified in this study are represented in the above concept map (Figure 1), which uses a series of circles to illustrate the themes with overlapping circles, representing links between the themes. At the center of the concept map is the dark orange circle, which represents the parent and the child with CP and the theme *My Child Doesn't Fit into the Box*. This theme encapsulates data that described how CP or disability makes a child different to the typical child that accesses healthcare and education systems. This difference consequently impacts upon the experience of finding adequate sleep solutions. Furthermore, this difference often created additional challenges due to systems not being designed to serve atypical children. The subsequent orange circles represent the ripple effects of the experience of sleep problems from the parent's perspective, which are linked to this perception of difference. The child's mismatch to the system and care leads to the requirement for parents to always watch out for their child (*A Mother's Ears are Always On*), including the work done overnight to care for their child, or to be awake with their child because of their child's sleep problems (*Sleep Disturbance is like Water Torture*). This then extends to sleep being a health issue that competes with many other, more urgent, health issues (*Sleep is one of Many Spot Fires, I Put it on the Backburner*). The final theme in the map (*Luck, Money or Jumping Up and Down*) illustrates the work that parents do to find sleep and other health-based solutions. That is, the central difference, the context of the sleep problem comorbidities, the inability to find clinicians familiar and skilled in CP care, and the increased care needs of CP, all impacted on sleep and seeking sleep solutions. The blue circles that fall on the opposite side of the map illustrate themes that are linked to effective solutions for sleep, or what the parents think may be effective solutions. These are placed in opposition to the themes that represent the experiences and challenges of systems, care or sleep problems. The individual themes will now be discussed with a focus on the participants' experience of seeking sleep care/sleep solutions with quotations from each theme.

### My Child Doesn't Fit Into the Box (My Child Is Atypical)

Overwhelmingly, the majority of parents shared that they thought their child with CP was atypical. This was the first and core theme identified, *My Child Doesn't Fit into the Box*. This theme encapsulates the frequently expressed perception that parents of a child with CP feel as if their child is different to other children. There were many ways in which this difference was described. It may have reflected the parent's own knowledge; not knowing what to expect or what is typical in a child with CP. Several of the parents discussed how their own unfamiliarity with CP and what to expect influenced their experience of their child and their expectations.

*I didn't know kids come out that early. Like I had no idea, totally no idea. So, for me, I was like, ‘Now what happens?’ And because I had nothing to relate it back to, ‘Oh so now this happens with him. Oh, ok’. I don't know other kids with CP*.

### A Mother's Ears Are Always on (Mothers Need to Be Vigilant)

This theme represents the vigilance of parents, usually mothers, in the overnight care of their children. This theme incorporates the data that describe both the overnight work as being the domain of the mother and the impact of that work on the mothers. The name of this theme is taken from a direct quote from Portia. However, it is recognized that it does apply to some fathers. The majority of the participants in this phase were mothers. While three fathers (Atlas, Orion, and Leo) were interviewed, participants Atlas and Orion articulated that their female partners (mothers) were the providers of overnight care, with Leo being the only father undertaking this role. However, his children did not have sleep problems. Of the interviews with mothers, only Pandora, Vela and Phoebe reported that their husbands shared the night-time care equally.

According to the mothers, providing overnight care was a natural expectation and an extension of their caring role. The majority of the mothers did not work, and this was the most common reason why their family accepted that the night-time work would be done by the mother. Many of the mothers described the need for their husbands to sleep so they could fulfill the requirements of paid work.

*I don't really sleep very well. It's just a habit now – it's just a routine for me… I'll start at about 11:30 to 12 o'clock, I'll get up the first time, and I'll turn her over, I'll just stretch her legs or she will stretch herself. Sometimes, I just check on her*.

### Sleep Disturbance Is Like Water Torture (Sleep Disturbance Has a Negative Impact)

The third theme to be identified was *Sleep Disturbance is like Water Torture*, which encapsulates the impact of poor sleep for the parents themselves, their child and their family life. All but three of the study participants discussed this theme. The naming of the theme came from Elara who, when asked about the impact of poor sleep, described disrupted sleep as, “*It's like Chinese torture, water torture.”* This theme directly addresses one of the research questions: ‘What is the impact of poor sleep on the parents and child?’ It covered how the parent described the impact of their child's sleep on themselves and what they perceived to be the impact of poor sleep on their child. Parents gave examples of how poor sleep impacted on their ability to participate in daytime activities with their family. This varied from changes in mood and patience through to opting out of joining in family activities.

*I'm just miserable, miserable, lack of energy. He also wakes his sibling up quite a lot, which means that she is feral. Generally, just feeling like crap and then not having that energy that maybe I would have to put the effort into trying… When you're running on empty, you just can't do it*.

### Sleep Is One of Many Spot Fires, I Put It on the Back Burner (Sleep Is Not a Priority)

Parents frequently talked about their child's sleep problems in the context of a more complex health profile. For all parents interviewed whose child had sleep problems, sleep was not the only health issue experienced by their child. This theme encapsulates data that conveyed where sleep sits as a priority for both parents and for clinicians (as reported by the parents). It was evident that good sleep was valued by the parents but not necessarily prioritized. Within the context of caring for a child with CP and the associated comorbidities and complexities, sleep was one of many problems on a list. It was clear from the interviews that sleep was not a common focus within the clinical setting. When asked, the majority of parents reported that they were rarely asked about their child's sleep in clinic appointments.

*Even though the sleep does go back into [*influencing*] the epilepsy and those sorts of things, I think because it's something that I can lifestyle-manage rather than, ‘We'll go in and have an operation and you'll sleep better.’ Like yeah. Cool. Look, if we don't sleep for two nights, it's not that big a deal. I'll just won't leave the house for two days and you can sleep during the day. Fine, whatever works… Because it's not a medical something, I feel like it's not as high on the priority list*.

### Luck, Money or Jumping Up and Down (Navigating Systems Is Challenging)

This major theme depicts the experience of parents interacting with systems including but not limited, to education systems, to healthcare in general and the NDIS. This theme includes *luck*: parents who described their experience of good care as “lucky”; *money*: parents who described electing to pay for private care (therapy, pediatricians, etc.); and *jumping up and down*: the advocacy and assertiveness required on behalf of the parents in order to navigate the system.

*Again, it's a private GP, a private paediatrician, and I think the only way you get these things done fast is if you pay for it, sadly, rather than going through the public system and waiting for your appointments and your referral. And I think that's probably why when we've had stuff and we've needed stuff, it's happened quite quickly. But we've paid for it*.

### There Is Never a Silver Bullet to Fix Things (There Isn't One Solution for Sleep)

Previously presented themes have highlighted the significant impact of sleep, the difficulty of navigating the system, and the difference created by CP, which is difficult to accommodate in a rigid system. Nine of the 16 parents reported they had obtained sleep solutions for their child. Four parents found pediatricians who helped improve their child's tone and in turn sleep. Five parents discovered solutions to help with behavioral management of sleep. One parent reported she used mostly homeopathy to treat her child. One mother was advised to prioritize her own sleep over the sleep of her child from early in life and followed this advice consistently. However, of those nine who had reported better sleep, five parents still experienced some sleep problems with their child. That is, better sleep was not always good sleep. What is clear from the data encapsulated in this theme is that the path to improved sleep is not linear. No parent reported a single treatment or intervention that solved their child's sleep problem; there is no ‘silver bullet’ to fix sleep. Indeed, many parents described trialing many unsuccessful sleep solutions.

*So, we just kept trying and trying and trying ourselves, and I would speak to the GP every now and again when it just got to a crisis level, and we'd just try something else and/or we'd move to a different room or we'd get a new bed, or various things like that [*happened*]… I read up about on the internet that really we just muddled through ourselves obviously*.

### Help: The Earlier the Better (Early Intervention Is Important)

A predominant theme that was identified in the interviews was the need for very early intervention in the form of knowledge about sleep. This theme includes what parents reported as being helpful or had helped their child's sleep problem. Overwhelmingly, when parents discussed what was or would have been helpful, the importance of early intervention, from when their child was young and newly diagnosed, was apparent. Many of the parents reported that early intervention had been helpful, or, on reflection, early intervention would have been beneficial. This help may have been specific to sleep or the provision of general information about what to expect in CP or disability.

*And then I thought like possibly, the hospital or the paediatrician or someone had said, ‘Hey, how's is sleep going?’ And I had answered. And then that might've been different, but yeah, I don't know. And I didn't really know who to ask*.

## Discussion

The findings of this study demonstrate that parents who seek help for sleep problems are often unsuccessful, either not receiving advice or treatment, or receiving advice or treatment that is not effective. This difficulty is punctuated by parents who feel that their child's needs do not match the systemic structures, or conversely, that the system does not match their child.

The parents in this study spoke of sleep not being a priority in the context of other more urgent health problems, and related that they felt clinicians rarely focused on sleep as a health issue. Several parents in this study reported that they had found successful sleep solutions and reported that they were under the care of pediatricians who applied a multi-layered approach to sleep problems in their child. They described a “trial and error” approach to solutions and sleep problems were revisited at subsequent appointments. Interestingly, only one family reported that they attended a sleep specific clinic. Some parents explained that finding sleep solutions was hard, and others just learnt to live with poor sleep. For the majority sleep issues are not a focus of clinical appointments as other, more urgent, health problems take priority.

In contrast to the findings of this study, McHugh (33) found that parents did not ask for sleep help and suggested that the context of the disability may have been a factor for parents not asking for help with sleep. McHugh's qualitative research suggested that the parents were tired, grieving or adjusting to their child's disability diagnosis, and this prevented them from asking for help. A study by Robinson and Richdale (34) reported that up to a third of parents with children with intellectual disability, including children with CP (6.5% of the study group), did not seek help for sleep problems. These two studies contrast with the current findings; in this study parents reported that they frequently asked for help with sleep from their healthcare team.

In the theme *Luck, Money or Jumping Up and Down*, an additional facet of seeking solutions was revealed: the work that parents, usually mothers, were required to do to navigate the system and find effective solutions. Australian studies have presented parents' general experience of navigating the healthcare system. These challenges have been reflected in the work of a Victorian based study by Bourke-Taylor et al. (35). They conducted a qualitative study of four mothers of children with a disability and four clinical professionals who work within the disability sector and Bourke-Taylor et al. (35) found that mothers reported a lack of cohesion between services and spent much time seeking resources, planning, locating and retaining services. This finding is supported by international studies (36). Within Bourke-Taylor's study, both mothers and professionals reported that the mothers were expected to constantly advocate for their children (35).

It is clear that the parents of children with CP are required to be tenacious care managers for their children in order to receive effective care. That is, there are significant gaps within the provision of health and disability care systems that parents are required to fill in order to avoid detrimental outcomes for their children. The theme *Help: The Earlier the Better* reiterates this concept of knowledge equating to empowerment.

There has been significant investigation into the experience of parenting, in particular mothering, children with a disability. A qualitative study from Canada (37) identified that parents of children with complex needs had little time for anything other than caring due to the demands of navigating services for their child. Those findings and other reported studies (36, 38, 39) resonate with the outcomes of the study reported in this paper. The complex context in which sleep problems are situated for these families and expectation of immense workload for parents is global. It might be argued that the volume of work that parents need to do every day to ensure good baseline care for their child is so high that a problem like sleep, for which there is little clinical urgency, could easily be deprioritized. If sleep problems are deprioritized, they likely go untreated, which in turn leads to chronic sleep disturbance and deprivation.

This study has demonstrated that sleep problems are multifactorial and complex and deeply entwined with many other aspects of disability: the parent child relationship, worry for a vulnerable child, the comorbidities of CP, and the non-linear nature of raising a child with a disability. Researchers need to design research projects that move beyond outcomes that simply count the number of sleep problems in study cohorts. The greater context of sleep problems needs to be considered, and a greater focus on meaningful interventions is required, in order to create clinical change for these families. Indeed, a study by Romeo et al. (40) concluded their paper with the declaration that a structured in-depth interview may have provided more accurate data to make better diagnoses, providing further support for the need for a multi-modal approach to data collection.

The findings of this research confirm that the experience of sleep problems in this cohort is often not linear. The experiences of the parents suggest that the complexity of disability in a child means that children often do not transition through sleep problems in the same way as a typically developing child might. Rather, parents described sleep problems improving and then worsening due to illness, surgical interventions, changes in family routine (for example, school holidays), or due to child or other life stressors and anxieties that impacted on sleep. There is no published literature which describes the experience of sleep problems and their solutions for children with CP over time. However, several studies support the finding that sleep problems are chronic (9, 34). This demonstrates that resolving sleep problems for children with CP may require repeated intervention over time. Any intervention for sleep in this group should incorporate this in its design.

To date, there is no published research regarding what sleep interventions are effective for children with CP, specifically. Clinicians and researchers may argue that diagnostic specific sleep interventions are not necessary, and that the significant overlap between neurodisability diagnoses may mean a transdiagnostic intervention can be used. Indeed, a recent systematic review by Rigney et al. (41). Reviewed the effectiveness of behavioral sleep interventions for children with neurodevelopmental disorders. The majority of the reviewed studies focused on autism spectrum disorder and attention deficit hyperactivity disorder, however, some studies included children with CP within sample groups. Rigney concluded that the current evidence suggests a transdiagnostic behavioral intervention is feasible. The findings of this study suggest that one singular sleep intervention is unlikely to address the needs of children with CP who have complex sleep problems. It is the contention of this paper that the management of sleep problems in children with CP needs to be, at least initially, individualized. As has been described by the parents in this study, an individualized approach to sleep problems allows for the complexity of the problems to be understood, and for treatment of comorbidities of CP that impact sleep to be remedied in the context of sleep problems.

## Limitations

The participants from this study were recruited from a previous quantitative phase. The overall demographics of that phase were parents with a higher than average education and socioeconomic status. It is likely that this research has not captured the experience of parents with less education or who are poorly resourced. The participants on this study were all residents of Victoria, Australia. Australia's health care system is different from state to state and the services and systems used in Victoria may not be generalizable throughout the country or internationally.

## Conclusion and Recommendations

This study plainly demonstrated that the experience of sleep in CP is not clear; it is a messy, convoluted and complex experience that is dependent on the resources of families, the individuals navigating the systems and their child's other health needs. The scattered nature of the circles in the concept map illustrates the complexity and the non-linear nature of sleep problems and sleep solution seeking that was demonstrated in this cohort of parents. Sleep and CP cannot be separated, and CP and sleep problems are enmeshed. This enmeshment is at a body systems level and from a healthcare system, family and parenting perspective. This study has captured rich and nuanced data that could not have been captured with quantitative methods. The findings demonstrates the many challenges parents face in sourcing effective sleep solutions for their child and the need for an individualized approach to sleep care. The promotion of sleep as a health priority needs to be elevated and more research is needed in order to determine how to educate both families and clinicians about the importance of sleep health. Consequently, targeted sleep education of clinicians who work with children with CP and their families' needs to be initiated. Allied health and nursing staff could have an important role in promoting and consulting on sleep health and this might be needed to create improvement in sleep for this cohort. This paper presents previously unknown data about the experience of seeking sleep care. This demonstrates the importance of consumer consultation in health services research. Any future research in this area should apply methods that enable consumer engagement.

## Data Availability Statement

The original contributions presented in the study are included in the article/supplementary material, further inquiries can be directed to the corresponding author/s.

## Ethics Statement

The studies involving human participants were reviewed and approved by the Royal Children's Hospital, Melbourne Human Research and Ethics Committee. The patients/participants provided their written informed consent to participate in this study.

## Author Contributions

SP conducted the research and wrote the paper as a Ph.D. candidate. FN, DR, and AH were Ph.D. supervisors for the project involved in all planning and implementation of the research as well as draft revision. SL also Ph.D. supervisor as above in addition to being the second researcher for the qualitative data analysis. All authors contributed to the article and approved the submitted version.

## Funding

This work was supported by the following scholarships awarded to SP: Australian Government Research Training Program Scholarship from the University of Melbourne, The Windermere Foundation Mary Patten Doctoral Scholarship in Health, The Royal Children's Hospital Foundation, The Vera Scantlebury Brown Child Welfare Scholarship, The Royal Children's Hospital Auxiliary, The League of Former Trainees.

## Conflict of Interest

The authors declare that the research was conducted in the absence of any commercial or financial relationships that could be construed as a potential conflict of interest.

## Publisher's Note

All claims expressed in this article are solely those of the authors and do not necessarily represent those of their affiliated organizations, or those of the publisher, the editors and the reviewers. Any product that may be evaluated in this article, or claim that may be made by its manufacturer, is not guaranteed or endorsed by the publisher.
